# Human pluripotent stem cell-derived cardiomyocytes for studying energy metabolism^☆^

**DOI:** 10.1016/j.bbamcr.2019.04.001

**Published:** 2020-03

**Authors:** Bärbel M. Ulmer, Thomas Eschenhagen

**Affiliations:** University Medical Center Hamburg-Eppendorf, Institute of Experimental Pharmacology and Toxicology, 20246 Hamburg, Germany; German Centre for Heart Research (DZHK), Partner Site Hamburg/Kiel/Lübeck, Hamburg, Germany

**Keywords:** ARVD, arrhythmogenic right ventricular dysplasia, ATP, adenosine triphosphate, CM, cardiomyocytes, EHT, engineered heart tissue, FFA, free fatty acid, gDNA, genomic, nuclear deoxyribonucleic acid, HF, heart failure, hiPSC, human induced pluripotent stem cells, IBMX, 3-isobutyl-1-methylxanthine, mtDNA, mitochondrial DNA, ROS, reactive oxygen species, Tissue engineering, Human induced pluripotent stem cells, Cardiac energy metabolism, Maturation, Cardiomyocytes, Developmental hypertrophy

## Abstract

Cardiomyocyte energy metabolism is altered in heart failure, and primary defects of metabolic pathways can cause heart failure. Studying cardiac energetics in rodent models has principal shortcomings, raising the question to which extent human induced pluripotent stem cell derived cardiomyocytes (hiPSC-CM) can provide an alternative. As metabolic maturation of CM occurs mostly after birth during developmental hypertrophy, the immaturity of hiPSC-CM is an important limitation. Here we shortly review the physiological drivers of metabolic maturation and concentrate on methods to mature hiPSC-CM with the goal to benchmark the metabolic state of hiPSC-CM against in vivo data and to see how far known abnormalities in inherited metabolic disorders can be modeled in hiPSC-CM. The current data indicate that hiPSC-CM, despite their immature, approximately mid-fetal state of energy metabolism, faithfully recapitulate some basic metabolic disease mechanisms. Efforts to improve their metabolic maturity are underway and shall improve the validity of this model.

## Highlights

• Dysregulated energy metabolism is a hallmark of cardiomyopathies and heart failure.
• Metabolism of human induced pluripotent stem cell-cardiomyocytes (hiPSC-CM) is fetal-like.
• Mimicking cues of postnatal development in vitro improves metabolism of hiPSC-CM.
• An adult state is not reached yet.
• But basic metabolic disease mechanisms can be recapitulated in hiPSC-CM.

## 1 The importance of the cardiac energy metabolism in healthy and diseased heart

The heart with its constant work load has a huge energy demand. In the healthy heart energy production meets the demand on a beat-by-beat basis due to a very high mitochondrial density and a flexible and efficient metabolism. During the development of heart failure (HF), in which the output of the heart at normal end-diastolic ventricular pressure is insufficient to meet the oxygen demand of the organisms, striking changes in cardiac energetics occur [1].

**Fig. 1:**
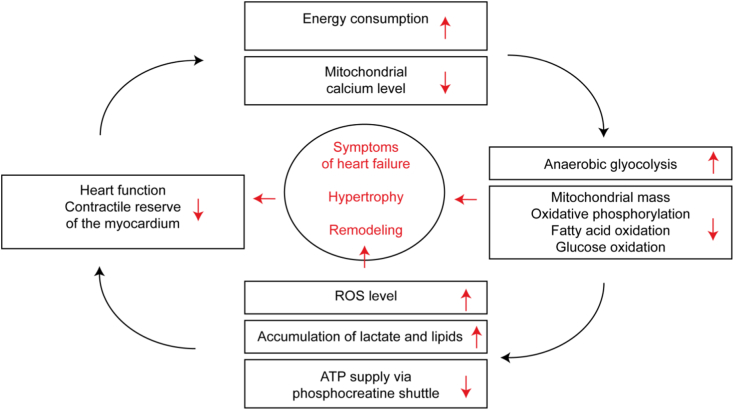
Hypothetical vicious cycle between heart function and cardiac energetics contributing to heart failure. Changes in cardiac metabolism and decreased heart function might not only correlate but interact in a negative feedback loop accelerating the development of heart failure.

While the normal heart generates over 90% of its energy from mitochondrial oxidative metabolism, the proportion of anaerobic glycolysis in adenosine triphosphate (ATP) production increases in HF with reduced or preserved ejection fraction. Overall mitochondrial oxidative metabolism and the transport chain activity are compromised [2]. Oxidation of glucose and/or β-oxidation of fatty acid decreases at least in the final stages of decompensated heart failure [3,4]. Mitochondrial mass and fusion of mitochondria are reduced [5]. The mitochondrial calcium level, which is supposed to balance ATP production with power consumption, is dysregulated [6]. As a result, the failing heart was named an “engine out of fuel” [7]. The most important hypotheses how changes in cardiac energetics affect cardiac output are (i) insufficient energy supply (ATP), (iii) reactive oxygen species (ROS) accumulation, (iii) lactate and (iiii) fatty acid accumulation (Fig. 1).

First, due to the inflexibility of energy metabolism seen in chronic HF, the ATP pool and the phosphocreatine/creatine energy shuttle are no longer sufficient to meet the needs of contractile work, initially under stress, and thus lead to less contractility [[8], [9], [10], [11]]. Second, a compromised electron transport chain activity leads to increased production of ROS, degeneration of the mitochondria, oxidation of important contractile proteins [12] and cell death [13]. Third, increased (anaerobic) glycolysis leads to lactate accumulation and acidosis, which in turn causes decreased calcium sensitivity of the contractile apparatus and thus reduced contractility [14]. Fourth, an imbalance of fatty acid uptake and consumption might result in fatty acid accumulation, lipotoxicity and cardiomyocyte (CM) death at least in diabetes-associated HF [8,15]. The changes in cardiac energetics are also discussed as one of the causes of activation of the fetal gene program [16]. Metabolic cardiomyopathies, in which heart failure is secondary to inherited defects of the energy metabolism [17], further underline how changes in the cardiac energy metabolism can be important drivers of cardiac remodeling.

Energy production is negatively affected as the degree of contractile dysfunction in HF increases and cardiac output decreases [18]. The reactivation of the fetal gene expression program results in a higher proportion of anaerobic glycolysis [19]. Stretching the heart worsens blood circulation and oxygen supply [18]. Finally, sarcomeres often work less effectively in the failing heart and consume more energy for the same contractile work than healthy CM. This is mirrored by mutations in sarcomere proteins, often observed in hereditary cardiomyopathies, which increase energy costs for force production [1]. In summary, disorders of energy metabolism can cause HF and the HF syndrome itself is characterized by humoral, structural and molecular changes that cause disturbances in energy metabolism, which might cause a vicious cycle (Fig. 1).

A better understanding and specific intervention into this cycle would be of great therapeutic interest. Many of the changes in energy metabolism observed in HF help to meet the energy demand during short term peak contractile work or insufficient oxygen supply. An example is anaerobic glycolysis which, in contrast to oxidative metabolism of fatty acids, produces ATP without O_2_ consumption and may thus be beneficial in ischemia. However, it might be detrimental in the long term as fatty acid oxidation is more effective in producing large quantities of ATP [5]. Thus, a simple distinction between maladaptive and beneficial alterations falls short [20]. Current heart failure therapy (e.g. with beta-blockers, ACE inhibitors, neprilysin inhibitors or ivabradine) aims at reducing the energy demand to alleviate the mismatch. Strategies specifically addressing the metabolic remodeling and/or mitochondrial stress could expand the therapeutic repertoire [21,22]. Interesting trials are on the way, e.g. with the tetrapeptide elamipretide (SS31) or MitoQ that increase mitochondrial energy or decrease ROS production [[23], [24], [25]], but no approved therapy directly addresses cardiac energetics. Several earlier trials that targeted energy metabolism, e.g. the ROS scavenger vitamin E or the PPAR gamma agonist rosiglitazone, had no beneficial effect or even increased death from cardiovascular causes [21,26,27].

One of the reasons for the difficulty in translating knowledge in this field could be the dominance of experimental rodent models. They were successful in establishing the effect of neurohumoral blockade and deciphering the genetic basis of HF, but may be less suitable for the study of cardiac energy metabolism. Mouse hearts beating with 600 beats per minute are already at their limits without much contractile, kinetic or metabolic reserve [28,29]. The mouse heart already has a high basal rate of glycolysis [30], reducing the room for increases in glycolysis in diseased conditions [31]. Ventricular human adult CM, on the other side, are difficult to obtain, hard to culture and often derived from end stage HF patients.

CM prepared from human induced pluripotent stem cells (hiPSC) could be an experimental model in which disease mechanisms can be experimentally deciphered in a human context. However, hiPSC-CM are immature directly after cardiac differentiation. Current protocols last between 10 and 21 days [[32], [33], [34]]. They consist of a first stage to induce mesoderm formation (Wnt activation) and a second stage to induce cardiac differentiation (Wnt inhibition), reproducing normal embryonic development [35]. However, given that the formation of cardiac chambers is completed at Carnegie stage 12, e.g. after the first 4 weeks of human development [36], it is clear that primitive hiPSC-CM directly after differentiation have a fetal phenotype. This raises the obvious question whether these primitive hiPSC-CM can faithfully reproduce the phenotype of adult CM, which is the main assumption underlying disease modeling. Increasing evidence suggests that further maturation can be achieved by relatively simple means. The necessary degree of maturity might vary depending on the question of interest, but investigation of CM energy metabolism, particularly of the mitochondria, probably requires a high degree of maturation.

## 2 Morphological changes of the heart after birth – perinatal developmental hypertrophy

The cardiovascular system and especially the heart undergo extensive growth and remodeling shortly before and in the first weeks after birth associated with anatomical, structural, functional and molecular maturation, called perinatal or postnatal developmental hypertrophy. These remarkable changes are driven by mechanical and hormonal factors and are crucial for *ex utero* survival, without the constant maternal supply of nutrients and oxygen and the removal of waste products through the placenta.

The circulatory system changes with the first breath. In fetal circulation, oxygenated blood bypasses the lungs through the foramen ovale between right and left atrium, and the ductus arteriosus between the pulmonary artery and the aorta. With the inflation of the lungs their resistance is drastically reduced while the systemic blood pressure increases. These changes lower the pressure in the right atrium and increase it in the left atrium, causing the foramen to become functionally closed. The oxygen content of the blood flowing through the ductus arteriosus increases, which leads to contraction of its smooth muscle cells and functional closure. Arterial pressure rises after removal of the low-resistance placental circulation. For the heart these changes mean a switch from a fetal serial to an adult parallel operation and a much increased work load especially of the left ventricle [10,[37], [38], [39]].

Along with these anatomical changes of the circulatory system and the higher work load, the weight of the heart, especially the left heart, increases. While the increase in heart mass during the embryonic and fetal period is predominantly achieved by cell division (hyperplasia), it is almost entirely due to an increase in size (hypertrophy) in post-natal stage. Neonatal CM are about 1000–1500 μm^3^ in size, adult CM between 20,000–25,000 μm^3^ [[40], [41], [42]]. Cells of such size need enough genetic material for their maintenance. In postnatal rodents, the majority of CM becomes binucleated. In humans, CM remain mostly mononucleated, but they become polyploid through DNA replication without karyokinesis or cytokinesis. Large cells also need a high degree of structural organization, and thus mature CM have a very dense, highly organized and almost crystalline cytoarchitecture, with densely packed sarcomeres alternating with strands of mitochondria and surrounded by a complex system of internal Ca^2+^ stores (the sarcoplasmic reticulum) and external membrane invaginations, the t-tubules [45]. The morphological development of the contractile apparatus and the electro-mechanical coupling elements in maturing CM is accompanied by a switch of sarcomere protein isoforms and ion channel composition. HiPSC-CM are much smaller (between 100 and 800 μm^3^; [43,44]) and much less organized than adult CM and thus resemble ~week 16 fetal human CM as discussed in many reviews [[45], [46], [47], [48]].

## 3 Changes in energy metabolism as hallmarks of perinatal maturation of CM – benchmarking hiPSC-derived CM

Developmental hypertrophy increases the contractile capacity of the heart, but at the same time the energy demand of each CM increases. This is met by molecular, structural and functional adaptations of the CM energy metabolism. Here we attempt to compare available data about the metabolic status of hiPSC-CM directly after cardiac differentiation, i.e. primitive hiPSC-CM, with hallmarks of metabolic maturation during developmental hypertrophy.

**Fig. 2:**
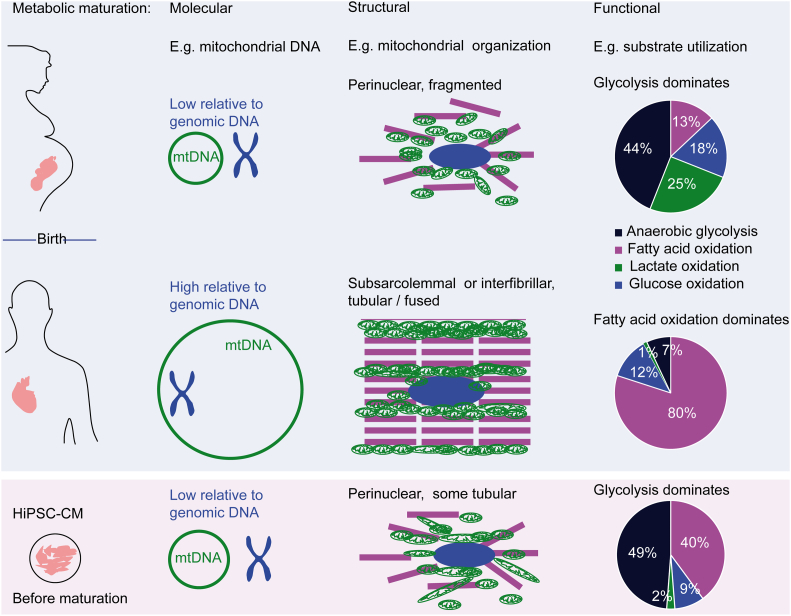
Benchmarking metabolic maturation status of hiPSC-CM. Hallmarks of maturation of cardiac energy metabolism during perinatal developmental hypertrophy and in hiPSC-CM. Examples include an increased ratio of mitochondrial DNA (mtDNA) to genomic DNA (gDNA), higher degree of subcellular organization and tubularization of mitochondria and switch of substrate utilization from anaerobic glycolysis to fatty acid metabolism [58]. Primitive hiPSC-CM depicts features of an immature energy metabolism with a low mtDNA/gDNA ratio, tubular, but perinuclear mitochondria and mostly anaerobic glycolysis.

Adapted with permission from [59].

The abundance of transcripts and proteins involved in energy metabolism is frequently evaluated as an easily accessible surrogate of the energy metabolism machinery in CM and hiPSC-CM. In normal cardiac development, maturation correlates with increased gene expression as well as protein abundance of several markers of mitochondrial biogenesis, tricarboxylic acid cycle (TCA) cycle and fatty acid metabolism. During developmental hypertrophy, the fraction of the mitochondrial proteome increases by a factor of 5 in adult compared to fetal heart samples, in turn cytosolic and nuclear protein abundance decreases [49,50]. Native embryonic CM and primitive hiPSC-CM exhibit lower transcription of TCA cycle or fatty acid β-oxidation marker proteins than adult CM, but higher than undifferentiated stem cells or mesodermal precursor cells [51]. Thus, the relative higher expression of these markers is a useful indicator, but reflects both CM differentiation and maturation. Another molecular marker reported in many studies is the abundance of mitochondrial DNA (mtDNA) normalized to the genomic, nuclear DNA (gDNA). Although the data scatters considerably between 1,000–40,000 [50]), probably mainly caused by differences in DNA extraction and qPCR procedures between studies, the mtDNA/gDNA ratio was 5-fold lower in fetal CM than in adult CM in the same study (2000 versus 10,000; [52]; Fig. 2). In human CM especially recombination-dependent replication initiation leads to replication of mitochondrial DNA [49,53]. Interestingly, the ratio of mtDNA to heart mass remains constant in humans between fetal and adult life [50], indicating that the abundance of mitochondria (=the capacity to generate ATP) is regulated according to the contractile mass and thus the energy demand. A decreased mtDNA copy number correlates with the risk for sudden cardiac death and cardiovascular disease [54,55]. In hiPSC-CM, mtDNA/gDNA was even ~50-fold lower than in adult heart tissue (250–600 vs. 1000–40,000, Fig. 2; [56,57]), indicative of major mitochondrial immaturity. The low mtDNA/gDNA ratio and thus mitochondrial density represent a major limitation of using hiPSC-CM as a model of adult CM to study energy metabolism.

On a structural level, mitochondria increase with developmental hypertrophy both in mass and organization, e.g. they develop higher cristae density and form more tubular networks. In addition, localization of mitochondria changes from predominantly perinuclear mitochondria to mostly subsarcolemmal mitochondria (SSM) and interfibrillar mitochondria (IFM) [50,60]. The more elongated IFM are spanning the sarcoplasmic reticulum and align with the sarcomere, where they supply ATP for the sarcoplasmic reticulum Ca^2+^ uptake and the contractile system, respectively. SSM are provide the energy for the transport of electrolytes and metabolites [49,61,62]. This differentiation is part of a process of the formation of energetic microdomains and the establishment of the phosphocreatine/creatine shuttle [63]. The maturation of mitochondrial morphology and localization was described as an active process, involving mitophagy of premature mitochondria mediated by Mitofusin-2 in vivo and during mESC differentiation to CM [64]. In hiPSC-CM, the majority of mitochondria is perinuclear, with different degrees of tubular networks [65,66]. The status of the phosphocreatine shuttle or the influence of mitochondrial fusion was not examined in hiPSC-CM so far.

Functionally, the most prominent hallmark of metabolic maturation is a switch in substrate usage from anaerobic glycolysis in fetal CM to oxidation of fatty acids as the dominant source of ATP production adult CM [67,68]. Most authors describe that anaerobic glycolysis is the dominant source of ATP production in primitive hiPSC-CM after differentiation and standard monolayer culture. Under our own conditions, monolayer-cultured hiPSC-CM produced almost half of the ATP from anaerobic glycolysis [59], others reported 74% [69]. Nose et al. recently examined the fatty acid/glucose uptake ratio with radioactive nucleoids and showed directly after differentiation a fatty acid uptake close to zero (in medium containing glucose, lactate and fatty acids), indicating a completely glucose-dependent metabolism [70]. Thus, primitive hiPSC-CM produce their ATP mostly by anaerobic glycolysis, reflecting an immature, fetal like functional cardiac metabolism. Still, hiPSC-CM are already flexible omnivores. hiPSC-CM can survive in medium containing fatty acid or lactate only [59], and glucose free, lactate-containing medium can be utilized to select CM from non-CM [51]. This reflects the well-known ability of CM to utilize high amounts of lactate even at the fetal or early newborn state [51,67].

In summary, primitive hiPSC-CM early after differentiation depict features of immature or fetal CM metabolism.

## 4 Methods to mature the energy metabolism mimicking physiological mechanisms

Approaches to overcome incomplete maturation of hiPSC-CM are based on two major hypotheses: (i) Identification of the molecular pathways that drive maturation of energy metabolism will allow specific interventions promoting maturation. Interesting examples of this strategy are repressing hypoxia-inducible factor 1α and lactate dehydrogenase A [71] or overexpressing micro RNA let-7 [72]. Both studies have shown to improve hiPSC-CM maturation including the energy metabolism, but are not the main scope of this review. (ii) In vitro culture conditions that mimic physiological growth stimuli in vivo will drive metabolic maturation [73,74]. The simplest approach in this direction is prolonged culture time, based on the fact that normal cardiac development also takes several months to reach an adult-like state [75]. However, even prolonged cell culture for 3 month failed to demonstrate an increase in mtDNA in hiPSC [76] and long culture time of hiPSC-CM before casting of engineered heart tissue (EHT) even decreased their maturity [77]. Thus, more complex strategies are needed.

Physiological cues that might be responsible for the postnatal switch in cardiac energy metabolism after birth are a change in energy substrate availability, higher work load, increased levels of several circulating hormones, higher oxygen supply and a change in cell composition of the growing heart (Table 1).

**Table 1:** Physiological cues that might drive metabolic maturation during developmental hypertrophy compared to hiPSC-CM maturation approaches. In vivo changes contributing to developmental hypertrophy are correlated with culture and tissue engineering protocols that investigated the impact on CM energy metabolism.

Changes during developmental hypertrophy		Approaches to mature hiPSC-CM	Effect on hiPSC-CM metabolism	Ref
Substrate availability	More free fatty acids, less glucose: milk as high fat low carbohydrate diet	Media composition with low/no glucose, fatty acid, lactose and/or galactose	Switch from anaerobic phosphorylation to oxidative respiration	[78] [79] [80]
Work load/mechanical load	Increased hemodynamic load, more contractile work of left ventricle	3D culture and tissue engineering	Increased mitochondrial biogenesis, structural improvements, and switch from anaerobic phosphorylation to oxidative respiration	[59] [69] [77] [81] [82]
Hormone status	Increase of T3, insulin, glucocorticoids and catecholamines	Supplementation of T3, insulin, glucocorticoids and cAMP-increasing drugs	T3: increased maximum respiratory capacity, no effect on mitochondrial biogenesis; Insulin/glucocorticoid/ IBMX: stimulated fatty acid oxidation.	[57] [65] [66] [83] [84]
Oxygen supply	Increase from 10 to 30 mm Hg fetal to 90–100 mm Hg postnatal	Culture under different ambient oxygen conditions	Not analyzed	
Cell composition	CM stop proliferation in contrast to non-CM, relative increase e.g. fibroblasts 2–3 fold	Different cell mixtures	Not analyzed	

### 4.1 Substrate availability

The energy supply through the placenta generates a constant glucose level close to the maternal blood level (around 5.5 mmol/l). In newborns an instant drop in blood sugar occurs, as milk is a high fat, low carbohydrate diet (around 3.5 mmol/l; [85]). Interestingly, insulin levels increase in neonates despite low glucose levels [86]. The average concentration of fatty acids in human plasma rises from less than 0.1 mmol/l in fetal to the adult range of 0.2 to 0.4 mmol/l [58,87] with high μM levels of bioavailable free fatty acid (FFA, 475 ± 252 μmol/l; [88]). Standard hiPSC-CM culture medium, e.g. RPMI1640 + B27, contains oleic acid, linoleic and linolenic acid in the low μM range (3–5 μmol/l). However, the amount of bioavailable FFA can be assumed to be in the nM range due to the low solubility of FFA and the high content of fatty acid binding albumin [89]. In addition, the glucose concentration of RPMI1640 + B27 amounts to 11 mM, largely exceeding the blood glucose concentration in infants and adults. It was therefore obvious to change the media composition by lowering glucose and providing substrates for oxidative metabolism [90]. Indeed, hiPSC-CM cultured in galactose- and fatty acid-containing medium produced their ATP by oxidative metabolism and not by glycolysis, mimicking a rather mature condition [78] and culture in fatty acid containing medium correlated with downregulation of fetal atrial natriuretic peptide [79]. Maturation medium with insulin and fatty acids but no glucose that forces hiPSC to maintain ATP synthesis by fatty acid β-oxidation, was shown to induce structural, molecular and electrophysiological maturation [80]. It remains to be explored whether glucose-free culture leads to a lasting switch in energy metabolism with higher oxidative phosphorylation capacity and higher mitochondrial density closer to adult CM. Interestingly, a recent study reported that after 4 weeks culture of hiPSC-CM under standard conditions the uptake of fatty acids increased from close to zero to 8-10fold of glucose uptake, similar to adult human cardiomyocytes that were investigated in parallel [70]. This is a striking difference, far more than expected just from prolonged culture. It is tempting to speculate that this metabolic maturation might be due to an initial 4–5 days culture in lactate containing, glucose free medium employed in this study.

### 4.2 Hormones

The best known hormone change around birth is the steep increase in total plasma 3,3′,5-triiodothyronine (T3), in sheep e.g. from 1 ng/ml to 4 ng/ml during the first hours after birth [91]. Normal values for total T3 levels in healthy humans are 0.80–2 ng/mL [92]. The relevant free concentration of T3 (the fraction not bound to blood proteins) is ~1000-fold lower than total T3 and amounts to 1.6–3.2 pmol/l (=0.65 pg/ml) in umbilical cord blood and raises quickly to 5.2–14.3 pmol/l on day 1–2 of life. Adult levels amount to 3.4–7.2 pmol/l [93]. Thyroid hormones are essential for heart development but also postnatal maturation where they surge as a response to cooling (reviewed in [94]). T3 is an essential component for serum-free medium of rat cardiac myocytes and hiPSC-CM [[95], [96], [97], [98]] and improve molecular and structural maturation [99,100], e.g. T3 stimulates the switch from fetal to mature titin isoforms [101]. High T3 supplementation with 20 ng/ml T3 added to RPMI-B27 (containing already 2 ng/ml T3, i.e. a hyperthyroid state) caused a 1.5-fold increase in maximal respiratory capacity of hiPSC-CM, suggesting some metabolic maturation. However, neither the mtDNA/gDNA ratio nor the mitochondrial volume fraction was increased [57].

A second postnatal adaptation is a surge in catecholamine secretion with a 3–10-fold increase in plasma epinephrine and norepinephrine levels around birth. Adrenergic stimulation promotes lipolysis and oxidation of fatty acids in fetal CM in a cAMP-dependent manner [84,102]. The increased adrenergic stimulation is further augmented by the ingrowth of sympathetic nerves into the heart, which starts already in the late fetal period in humans (compared to strictly postnatally in rodents; [103]), but probably mature in the post-natal period [104]. It is not known yet whether sympathetic stimulation or co-culture with sympathetic neurons improves the (metabolic) maturity of hiPSC-CM. Glucocorticoid blood levels already rise strikingly in the last weeks before birth, important for maturation of the organs. Most prominent is their role in lung development, but the right level of glucocorticoid is also mandatory for normal heart development. Glucocorticoids induce key regulators of mitochondrial biogenesis such as PGC1α (peroxisome proliferator-activated receptor gamma coactivator 1-α) and can increase mitochondrial oxidation of both glucose and fatty acids [105,106]. Accordingly, some hiPSC-CM maturation protocols combine glucocorticoid (dexamethasone), insulin and 3-isobutyl-1-methylxanthine (IBMX, a phosphodiesterase inhibitor leading to increased cAMP levels [66,84]. This intervention increased oxidative metabolism and expression of PPARα. hESC-CM cultured with dexamethasone, T3, and insulin-like-growth factor 1 showed increased contractile force generation and improved bioenergetics with higher PGC-1α and PGC-1β mRNA levels and increased utilization of both glucose and fatty acids [83]. Another humoral change after birth is an increase in angiotensin II and aldosterone [104], but their effect on hiPSC-CM is unknown.

### 4.3 Increased work load

After birth the left ventricle faces a much higher work load resulting from the remodeling of the cardiovascular system as discussed above. To mimic the physiological 3-dimensional (3D) tissue environment and the increased afterload of the developing heart (i) hiPSC-CM are increasingly cultured as 3D tissue and (ii) exposed to mechanical load. Multiple ways to culture hiPSC in 3D and expose engineered tissues to mechanical load have been developed over the past decade, mainly based on original hydrogel protocols developed with chicken [104] and rat cardiac cell-derived EHT [88]. This culture form led to improved maturation in terms of ultrastructural organization, contractility and electrophysiological properties (reviewed in [107,108]).

A few studies also investigated the impact of 3D culture on CM energy metabolism. Culture of aggregates of hiPSC-CM without scaffolds is the simplest way of 3D culture, which led to lower expression of markers of glycolysis and lipogenesis, while components of the TCA cycle were higher expressed compared to 2D culture. Accordingly, the contribution of glycolysis to the total ATP production decreased from 68% to 49% [69], indicating metabolic maturation.

**Fig. 3:**
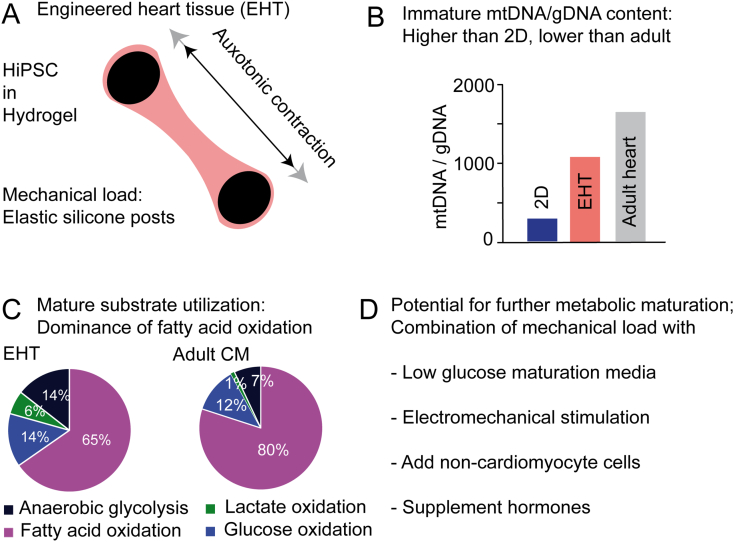
Metabolic maturation in engineered heart tissue (EHT). (A) Schematic depiction of EHT technology. (B, C) Molecular (B) and functional (C) investigation of the energy metabolism depicts different degrees of maturity. Adapted with permission from [59] (D) Outlook: combining different cues for improved maturation.

Hydrogel-based EHTs are nowadays the most frequently used cardiac tissue engineering method. hiPSC-CM are mixed with an initially liquid hydrogel that solidifies in casting molds around a mechanic support that provides the tissue with mechanical load. By utilizing elastic materials like silicone posts or elastic suspension EHTs can perform a physiological mode of contractile work, namely auxotonic contraction against a mechanical load (Fig. 3A; reviewed in [107]). Of note, the work of hiPSC-CM in EHTs in vitro is considerably lower than the work of the heart muscle in vivo. We showed that cultivation of hiPSC-CM in 3D EHT format increased mitochondrial mass and protein abundance compared to batch- and time-matched 2D cultured hiPSC-CM. The ratio of mtDNA to gDNA was almost 3-fold higher in 3D than in 2D cultured hiPSC-CM, but still considerably lower than in human heart samples assayed in parallel (2D 343; 3D 1024; human heart 1501; Fig. 3B; [59]). Functionally, 3D-hiPSC-CM in EHTs derived more ATP from the oxidation of glucose (14% compared to 9% in 2D), lactate (6% compared to 2% in 2D), and fatty acid (60% compared to 40% in 2D) and less from anaerobic glycolysis (14% compared to 49% in 2D), approaching values published for adult cardiac metabolism (Fig. 3C). Pharmacological reduction of contractile force blunted the increase in mitochondrial mass and DNA in EHTs, indicating that the higher contractile work in 3D tissues contributes to this metabolic maturation [59].

### 4.4 Oxygen levels

An additional drastic change after birth is the oxygen supply. During early embryonic development, (arterial) blood oxygen concentration amounts to less than 10 mm Hg, reaches 10–20 mm Hg (~2%) before birth and 90–100 mm Hg (~15%) after birth [109]. Cell cultures are usually cultured under ambient oxygen, i.e. 160 mm Hg (~21%). While this suggests standard culture conditions to be in fact hyperoxic, 3D tissues rely on diffusion to a much higher degree than standard 2D monolayers, and the oxygen supply in the middle of the tissues might still be a limiting factor, as oxygen concentrations of 40% increased force in neonatal rat tissue [110]. This stimulated the development of endothelial cell or scaffold based vascularization methods [111,112]. However, no data on the effect of different oxygen supplies on metabolic maturation of hiPSC-CM is available.

### 4.5 Cell-cell interaction

While cardiomyocytes occupy most of the cardiac cell mass, non-cardiomyocytes constitute 70–75% of the cells in the adult heart. Most abundant are endothelial cells with over 60% of non-CM, fibroblasts with 20%, leukocytes, smooth muscle cells, and pericytes [113]. Fibroblast number increases in relation to CM 2–3 fold after birth. Addition of non-CM, especially fibroblasts and endothelial cells, was shown to improve structural and molecular maturation of hiPSC-CM as well as contractility of 3D tissues [32,[114], [115], [116], [117], [118]]. However, to our knowledge the impact of co-culture of non-CM with hiPSC-CM on metabolic maturation was not assessed to date.

### 4.6 Combination of different maturation approaches

3D culture easily allows the further addition of maturation signals, as discussed already regarding cell composition and hormone supplementation. Another possibility is electromechanical stimulation that was shown to improve structural, molecular and electrophysiological maturation. Electrical stimulation of hiPSC-EHTs improved mitochondrial cristae density and organization but did not reach adult human cardiomyocytes [82]. In a similar model, prolonged high frequency stimulation increased expression of genes involved in mitochondrial biogenesis and metabolism and induced the mitochondrial volume fraction to ~30%, matching that of adult human CM [77]. Seahorse measurements portrayed both an increase in oxidative capacity and in glycolytic capacity [77]. Another possibility is the combination of maturation media with 3D tissue culture. Mills et al. combined microtissue organoid culture of hiPSC-CM with a maturation medium containing low glucose and the fatty acid palmitate, but no insulin. The maturation medium not only improved contractile function and other maturation markers, but also induced the postnatal switch from carbohydrate to fatty acid substrate utilization [81,90].

Taken together, the data indicate that the combination of mechanical, electrical, nutrient and hormone stimuli that is mimicking the different cues driving postnatal developmental hypertrophy results in more mature hiPSC-CM based models of cardiac energetics.

## 5 Phenotyping energy metabolism in hiPSC-CM based disease models

While many groups including ours strive to improve the maturity and thereby the quality of hiPSC-CM, they are already utilized for disease modeling. To cite George Box, “The most that can be expected from any model is that it can supply a useful approximation to reality: All models are wrong; some models are useful”. A litmus test how useful hiPSC-CM are is the reliability with which typical mitochondrial abnormalities known from inherited diseases can be reproduced in patient- and disease-specific hiPSC lines (Table 2). Soon after the seminal discovery of Yamanaka [119] the first studies utilized hiPSC-CM from patients with inherited cardiac diseases like Long-QT or LEOPARD Syndrome for disease modeling [120,121]. Today, hiPSC-CM have been generated for almost all heart diseases with (likely) monogenic inheritance, mostly ion channel diseases (reviewed in [122]) and structural cardiomyopathies [123]. While data still scatter significantly, some differences between healthy and diseased hiPSC-CM appear to faithfully recapitulate hallmarks of hypertrophic and dilated cardiomyopathy, e.g. lower force in DCM-CM and larger cell size in HCM-CM (reviewed in [123]).

**Table 2:** Disease modeling utilizing hiPSC-CM, restricted to studies describing changes in energy metabolism.

Condition	Reported metabolic abnormalities in diseased hiPSC-CM	Ref.
Barth syndrome	Fragmented mitochondria, elevated basal oxygen consumption rates and impaired electron transport chain	[65] [124]
Pompe disease	Degenerated mitochondria, lower oxygen consumption rate, accumulation of glycogen	[[125], [126], [127]]
Friedreich's ataxia	mtDNA depletion, mitochondria network disorganization and lower level of respiratory chain proteins	[128]
Arrhythmogenic right ventricular dysplasia (ARVD)	PPARγ over-activation and lipid accumulation; diseased hiPSC-CM directly transdifferentiate into adipocytes	[66] [84] [[129], [130], [131]]
Danon's disease	Reduced mitophagic flux resulted in mitochondrial fragmentation	[132] [134]
Hypertrophic cardiomyopathy	Abnormally high metabolic respiration rate	[44]

Here we restrict ourselves to the discussion of the few studies that found differences in mitochondria or cardiac energy production between patient-specific hiPSC-CM and (gene edited) control cell lines (Table 2).

Mitochondrial cardiomyopathies were among the first diseases studied in hiPSC-CM. Wang et al. studied cells from patients with Barth syndrome (BTHS), a mitochondrial disorder caused by mutations of the gene Tafazzin (TAZ). They described more fragmented mitochondria in 2D cultured hiPSC-CM BTHS, elevated basal oxygen consumption rates and impaired electron transport chain activity [65]. ATP levels decreased in glucose-free medium, which was associated with abnormal sarcomere organization and decreased contractility measured in muscular thin sheets. Interestingly, the sarcomere organization and function could be rescued with the ROS scavenger MitoTEMPO, a finding that provided evidence for a current trial with elamipretide [133]. hiPSC-CM from patients with Pompe disease, an autosomal recessive glycogen storage disorder due to mutations in the gene for acid β-glycosidase, exhibited degenerated mitochondria, lower oxygen consumption rate and higher glycogen content. Treatment with l-carnitine reverted the phenotype partially [125]. Accumulation of glycogen was confirmed in two other studies [126,127]. A third example is Friedreich's ataxia, a recessive neurodegenerative disorder caused by mutations (GAA triplet expansion) in the gene encoding the mitochondrial protein frataxin. hiPSC-CM revealed mtDNA depletion, mitochondria network disorganization and lower level of respiratory chain proteins succinate dehydrogenase and cytochrome *c* oxidase [127].

In addition to these metabolic cardiomyopathies, where a disturbed energy metabolism is the primary cue underlying structural or functional cardiac changes, inherited structural cardiomyopathies also often exhibit metabolic disarrangements. Arrhythmogenic right ventricular dysplasia (ARVD) patient specific hiPSC-CM depicted abnormal PPARγ over-activation and lipid accumulation [66,84,129,130]. Culturing these cells in a lipogenic medium (+insulin, dexamethasone, IBMX, rosiglitazone and indomethacin) caused even more lipid accumulation and exaggerated rates of apoptosis [66]. Another study showed that ARVD-hiPSC-CM directly transdifferentiate into adipocytes when cultured in a similar lipogenic medium (+insulin, dexamethasone, IBMX and indomethacin), suggesting a possible mechanism of the well-known fibroadipocytic replacement of cardiac myocytes in ARVD [131].

Danon's disease is characterized by skeletal muscle weakness and severe cardiomyopathy caused by LAMP-2 mutations resulting in defective autophagic flux. Danon's disease derived hiPSC lines showed that reduced mitophagic flux resulted in mitochondrial fragmentation, associated with contractile dysfunction and apoptotic cell death [132,134]. Apoptotic cell death was prevented by scavenging ROS with the clinically approved antioxidant *N*-acetylcysteine, suggesting that the reduced mitophagy leads to accumulation of defective, ROS-producing mitochondria and this plays an important role in the pathogenesis of the disease.

Energetic imbalance can not only be caused by defective ATP generation as discussed above, but also by inefficient ATP usage, particularly in highly ATP-consuming processes such as crossbridge cycling. This mechanism may be part of the pathogenesis of sarcomeric cardiomyopathies (reviewed in [1,123]). A recent study produced 11 variants of HCM-related mutations in β-myosin heavy chain with CRISPR/Cas9 editing in 3 independent hiPSC lines and examined hiPSC-CM both in 2D and EHTs. One of the key findings was an abnormally high metabolic respiration rate in the diseased lines. The data suggest inefficient ATP usage as the underlying cause and nicely support the energy depletion model [44].

Taken together, these studies suggest that the metabolic phenotype or at least some key features of mitochondrial cardiomyopathies can be reproduced in hiPSC-CM despite the immaturity. A caveat, though, is that nobody systematically determined the normal metabolic variability of “control” hiPSC-CM and tried to prospectively identify diseased from healthy lines in a blinded manner.

## 6 Conclusion and future directions

A disturbed energy metabolism might accelerate or even cause the development of heart failure in many cardiac diseases, and thus is a potential target for pharmacological therapies. In principle, patient-specific hiPSC-CM are able to recapitulate these processes. However, with the current status of limited metabolic maturation in hiPSC-CM, it is likely that only changes with a high effect size can be detected reliably. Diseased CM and immature CM are in many aspects indistinguishable, for example with respect to mitochondrial organization, oxidative capacity or substrate usage. To provide meaningful results that reflect the disease-causing mechanisms in human patients, this limitation must be considered. Depending on the question, a maturation phase might be mandatory. Very likely, the combination of medium optimization and adapted mechanical loading mimicking different cues occurring during developmental hypertrophy will create a physiological environment that allows maturation of hiPSC-CM. Interventions targeting molecular pathways that drive maturation of energy metabolism could further accelerate these maturation processes. This will allow in future to investigate more subtle changes in a patient-specific way to pave the way for the development of new treatment options.

## Conflict of interest

TE is founder and co-owner of EHT Technologies.

## Transparency document

Transparency document. Image 1
